# Multistrain models predict sequential multidrug treatment strategies to result in less antimicrobial resistance than combination treatment

**DOI:** 10.1186/s12866-016-0724-5

**Published:** 2016-06-23

**Authors:** Amais Ahmad, Camilla Zachariasen, Lasse Engbo Christiansen, Kaare Græsbøll, Nils Toft, Louise Matthews, John Elmerdahl Olsen, Søren Saxmose Nielsen

**Affiliations:** Department of Large Animal Sciences, Faculty of Health and Medical Sciences, University of Copenhagen, Grønnegårdsvej 8, DK-1870 Frederiksberg C, Denmark; Department of Veterinary Disease Biology, Faculty of Health and Medical Sciences, University of Copenhagen, Frederiksberg C, Denmark; Department of Applied Mathematics and Computer Science, Technical University of Denmark, Richard Petersens Plads, 2800 Lyngby, Denmark; National Veterinary Institute, Section of Epidemiology, Technical University of Denmark, Bulowsvej 27, DK-1870 Frederiksberg C, Denmark; Boyd Orr Centre for Population and Ecosystem Health, Institute for Biodiversity, Animal Health and Comparative Medicine, College of Medical, Veterinary and Life Sciences, University of Glasgow, Glasgow, UK

**Keywords:** Antimicrobial resistance, Ampicillin, Tetracycline, Pharmacodynamic, Dosing strategies, Pig, Bacterial growth

## Abstract

**Background:**

Combination treatment is increasingly used to fight infections caused by bacteria resistant to two or more antimicrobials. While multiple studies have evaluated treatment strategies to minimize the emergence of resistant strains for single antimicrobial treatment, fewer studies have considered combination treatments. The current study modeled bacterial growth in the intestine of pigs after intramuscular combination treatment (i.e. using two antibiotics simultaneously) and sequential treatments (i.e. alternating between two antibiotics) in order to identify the factors that favor the sensitive fraction of the commensal flora.

Growth parameters for competing bacterial strains were estimated from the combined in vitro pharmacodynamic effect of two antimicrobials using the relationship between concentration and net bacterial growth rate. Predictions of in vivo bacterial growth were generated by a mathematical model of the competitive growth of multiple strains of *Escherichia coli.*

**Results:**

Simulation studies showed that sequential use of tetracycline and ampicillin reduced the level of double resistance, when compared to the combination treatment. The effect of the cycling frequency (how frequently antibiotics are alternated in a sequential treatment) of the two drugs was dependent upon the order in which the two drugs were used.

**Conclusion:**

Sequential treatment was more effective in preventing the growth of resistant strains when compared to the combination treatment. The cycling frequency did not play a role in suppressing the growth of resistant strains, but the specific order of the two antimicrobials did. Predictions made from the study could be used to redesign multidrug treatment strategies not only for intramuscular treatment in pigs, but also for other dosing routes.

**Electronic supplementary material:**

The online version of this article (doi:10.1186/s12866-016-0724-5) contains supplementary material, which is available to authorized users.

## Background

Use of antimicrobials is often essential in modern livestock production in order to ensure a sufficient level of animal health and welfare, yet both animal and public health concerns stress the importance of treatment protocols that minimize the emergence of resistant bacteria. Concern over public health is linked to the emergence of resistance in zoonotic bacteria as well as in commensal bacteria, which may transmit resistance genes to bacteria in the human gut through the food chain [1].

In 2012, the Danish surveillance scheme on antimicrobial resistance (DANMAP) showed that 32 % of the *Escherichia coli* isolates from pigs were multi-resistant. The majority of these (78 %) were co-resistant to ampicillin, sulfonamide and streptomycin, and more than half of these (55 %) were also resistant to tetracycline [2]. In order to reduce the level of multi-resistant strains, treatment protocols based on multiple drugs are possible [3], and multidrug therapy has increasingly been used worldwide to fight infections caused by bacteria resistant to more than one antimicrobials [4–6].

Careful investigation of treatment strategies in multidrug treatment is required in order to evaluate the effect on the commensal flora, as the outcome may depend on cycling strategy [3], susceptibility levels of bacteria [7], and the nature of interaction between the antimicrobials [8]. These interactions are generally classified as synergistic, antagonistic or additive [9]. While antagonistic effects (i.e. the combined treatment efficacy of two drugs is less than the effect of the individual treatments) is unwanted from a treatment point of view, studies with doxycycline (an important tetracycline drug used in both animal and human medicine) have shown that combination treatment with antagonistic drugs (ciprofloxacin in low concentrations) favored sensitive *E. coli* over tetracycline resistant *E. coli* in competition experiments [8]. The aim of the current study was to evaluate this observation with another typical drug class that is antagonistic to tetracycline (β-lactams) and to elucidate whether it was dependent upon the mode of treatment (sequential versus combined treatment). The study by Chati et al, [8] was conducted as a competition assay between two isogenic strains, though in the intestine a much more complex competition between many strains occurs. For example, a recent study showed that nursery pigs in intensive pig production in Denmark may harbor many different *E. coli* strains [10]. Therefore, we extended a growth model that we previously developed to study the growth dynamics of multiple *E. coli* strains in a pig following intramuscular (IM) tetracycline treatment [11] to include combination treatment with ampicillin.

## Methods

### In vitro growth of *E. coli* strains under a combination of drugs

Growth curves for the combined effect of tetracycline and ampicillin on ten selected *E. coli* strains were performed using the automated microbiology growth curve analysis system BioScreen C™ (Oye Growth Curves Ab Ltd, Finland) Additional file 1: Figure S1. These ten strains were selected among the fifty *E. coli* strains in order to obtain all possible phenotype combinations and respective resistance levels. Where, fifty *E. coli* strains were randomly selected among 160 porcine indicator *E. coli* isolates from the Danish Integrated Antimicrobial Resistance Monitoring and Research Program (DANMAP) in 2010 [12]. The 50 isolates were subcultured, and the susceptibility of strains to tetracycline and ampicillin was tested using a broth microdilution susceptibility test, following the Clinical and Laboratory Standards Institute (CLSI) guidelines [13]. Results on tetracycline susceptibility have previously been reported [11]. A cut-off value of 8 μg/ml between susceptible and resistant *E. coli* strains for both tetracycline and ampicillin was adopted from DANMAP 2010. The ten selected strains were divided into the four groups, comprising of strains resistant to tetracycline only (R_T_S_A_), resistant to ampicillin only (S_T_R_A_), resistant to both (R_T_R_A_) and susceptible to both (S_T_S_A_) with a total of three, two, one and four strains, respectively (Table 1). Four static concentrations of each antimicrobial, (0, 0.25, 0.5, 16) μg/ml for tetracycline, and (0, 1, 4, 16) μg/ml for ampicillin, were used in a four-by-four array. All isolates were additionally grown in MH-2 media without antibiotics. The BioScreen was set to 18 h incubation at 37 °C with continuous (250 rpm) shaking, and optical density (OD) at 600 nm measured every 5 min. All experiments were performed in biological triplicate.

**Table 1:** Estimated growth parameters and prior growth parameters of ten *E. coli* strains used in the model for competitive growth

Strain	MIC-tet μg/ml	MIC-amp μg/ml	Combination	Parameter Coefficients					Priors			
				α_max_	EC_50tet_	γ_tet_	EC_50amp_	γ_amp_	EC_50tet_	γ_tet_	EC_50amp_	γ_amp_
A	2.00	1.00	S_T_S_A_	1.93	1.14	8.35	0.81	2.05	1.14	8.35	0.67	1.92
B	128	4.00	R_T_S_A_	1.93	74.03	11.24	5.21	7.03	74.03	11.24	2.53	5.00
C	0.50	1024	S_T_R_A_	1.81	0.24	1.35	440	5.14	0.15	1.87	440.7	5.14
D	512	1024	R_T_R_A_	1.77	187	3.99	400	5.00	186.9	3.99	400	5.00
E	64	8.00	R_T_S_A_	2.03	67.5	8.23	4.95	4.84	67.46	8.23	2.85	3.96
F	0.50	4.00	S_T_S_A_	2.05	0.32	2.42	4.70	5.03	0.23	2.80	2.33	4.00
G	0.50	8.00	S_T_S_A_	1.93	0.24	1.73	3.59	4.43	0.18	2.24	3.62	4.42
H	16	2.00	R_T_S_A_	1.73	9.24	13.95	1.33	1.53	9.24	13.95	1.29	5.00
I	0.50	256	S_T_R_A_	1.84	0.20	1.26	151.5	5.00	0.14	1.72	151.5	5.00
J	0.25	2.00	S_T_S_A_	1.76	0.12	1.24	0.46	2.64	0.11	1.83	0.64	2.32

### Growth rate estimation

The BioScreen raw data were extracted in Microsoft Excel. OD values of blank samples were subtracted from sample OD values at the respective time points before the data were analyzed using R (version 3.0.1 for Windows) [14]. The effect of combined antimicrobial concentrations on the growth of *E. coli* was assessed from the net growth rate (*μ*) of the strains with 16 different combinations. As a linear relationship between colony forming units (CFU) and OD is only valid for low cell concentration and this relation becomes unreliable above a certain critical value [15], an OD of 0.1 was taken as a maximum reliable value in this study. This value was used as the threshold, and therefore only the exponential growth part of growth curves below this cut-off was used for the model fit. The following model equation was used:

1 $$ {Y}_t=\theta {e}^{\mu t}+\beta +{\varepsilon}_t $$

with the parameters *Y*_*t*_: the OD value, *θ:* the initial OD value at time zero, *μ*: the growth rate, *β*: an offset variable for the adjustment of *θ,* and*ε*_*t*_: a normal error with mean zero and constant variance*σ*^2^; i.e., *ε*_*t*_ = N(0, *σ*^2^). Growth rates for the ten *E. coli* strains at each combination were estimated by fitting the model (equation 1) to growth curves over 18 h using a nonlinear least square algorithm nls() function of the R software [14].

### Estimation of growth parameters and evaluation of drug interaction

The relationship between antimicrobial concentrations and net bacterial growth rates under combined concentrations of tetracycline and ampicillin was analyzed using:

2 $$ \alpha \left({c}_a,{c}_b\right)={\alpha}_{\max}\left(1-\frac{{c_a}^{\gamma_a}}{E{C_{50a}}^{{}^{\gamma_a}}+{c_a}^{\gamma_a}}\right)\left(1-\frac{{c_b}^{\gamma_b}}{E{C_{50b}}^{{}^{\gamma_b}}+{c_b}^{\gamma_b}}\right) $$

**Fig. 1:**
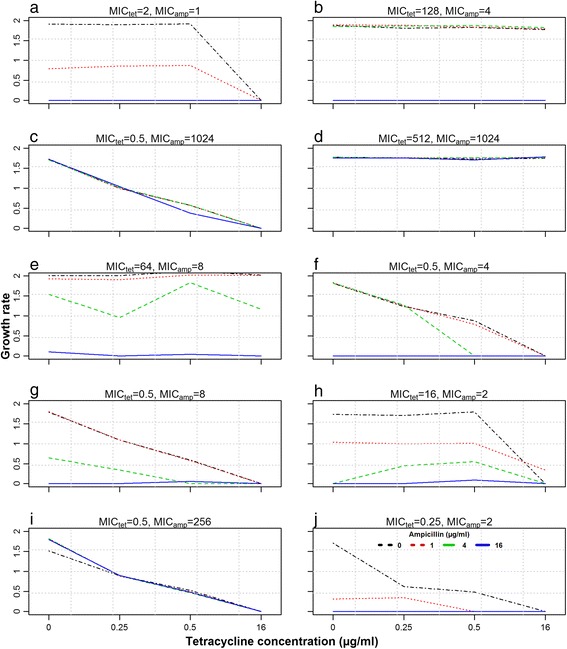
Interaction plots of the effect of tetracycline and ampicillin concentrations on bacterial growth. Each box represents one strain, with the x-axis as tetracycline concentrations and different lines representing ampicillin concentrations

Where*α*(*c*_*a*_, *c*_*b*_): the net bacterial growth rate with the combination of tetracycline concentration*c*_*a*_and ampicillin concentration*c*_*b*_,*α*_max_: the bacterial growth rate in the absence of both drugs, *EC*_50_: the concentration at which the drug effect is reduced to 50 %, and γ: the Hill coefficient, which is the measure of the steepness of the sigmoid relationship between concentration *c* and the growth rate at concentration *c*. The subscript *a* refers to tetracycline, and *b* refers to ampicillin, in*EC*_50_, γ and *c*. We had a total of five parameters to estimate for each strain (*α*_max_, *EC*_50*a*_, *EC*_50*b*_, *γ*_*a*_, *γ*_*b*_). Growth rates in triplicates derived from the exponential growth model (equation 1) were plotted against the concentration ranges of both tetracycline and ampicillin (Fig. 1) and fitted to the model (equation 2) for each of the ten *E. coli* strains, using a nonlinear minimizing routine nlminb() of software R [14]. Strains with a minimum inhibitory concentration (MIC) value greater than the concentration ranges of the drugs used for in vitro growth curves did not show any decrease in growth rates (Fig. 1b, d). In such cases, we did not have any pharmacodynamic information, and a prior information term ${\displaystyle \sum {\left(\frac{p-{p}_{prior}}{p_{prior}}\right)}^2}$ (from previously estimated growth parameters of these strains for tetracycline [11] and ampicillin treatment (Ahmad A, Zachariasen C, Graesboll K, Christiansen LE, Toft N, Matthews L, et al. Modeling the growth dynamics of multiple Escherichia coli strains in the pig intestine following intramuscular ampicillin treatment. submitted) was added to the residual sum of squares of the model equation for all parameters except for *α*_max_ in order to set up a function for optimization of the non-linear relation (equation 2). Inclusion of this term insured the convergence of the function, even in the absence of sufficient information in a relationship of growth and drug concentrations. Outliers from triplicates at each combined concentration were removed based on set criteria (e.g. if the maximum absolute residual value from model fit is greater than the absolute residual difference between the other two replicates) before the final fit. The interaction of two drugs was analyzed using the residual structure of the fitted model.

### In vivo pharmacokinetics

Data for the changing plasma concentrations following IM treatment of tetracycline and ampicillin in pigs were obtained from literature [16, 17]. Data were fitted to a two-compartmental model to estimate absorption, distribution and elimination rates for each of two drugs (data not shown). Based on these rates, plasma concentration profiles of tetracycline and ampicillin were generated and used simultaneously and sequentially. The treatment duration was set to a maximum of 6 days.

### Mathematical model

The model previously developed to describe the growth of multiple strains of *E. coli* following IM treatment with tetracycline [18] was extended to include the growth of ten *E. coli* strains in a pig, following multiple IM treatments of tetracycline and ampicillin.

The changes in the bacterial counts of individual bacterial strains in a pig was modeled using estimated growth parameters from the pharmacodynamic (PD) model (equation 2) combined with the in vivo drug profiles, using an ordinary differential equation:

3 $$ \frac{d{N}_i}{dt}=\alpha \left({c}_a,{c}_b\right)\left(\frac{N_{\max }-{N}_i}{N_{\max }}\right)\left(\frac{N_{\max }-\varSigma {N}_i}{N_{\max }}\right){N}_i-\varphi {N}_i $$

with $\frac{d{N}_i}{dt}$: the change in the bacterial counts *N*_*i*_ of strains *i*, *α*(*c*_*a*_, *c*_*b*_): net bacterial growth rate at the combination of tetracycline concentration *c*_*a*_, and ampicillin concentration*c*_*b*_as given in equation 2,

$\left(\frac{N_{\max }-{N}_i}{N_{\max }}\right)\left(\frac{N_{\max }-\varSigma {N}_i}{N_{\max }}\right){N}_i$: density-dependent growth limitations, which depend on the carrying capacity, *N*_*max*_, and the total bacterial counts summed over all competing strains in the pig, *ΣN*_*i*_, *φN*_*i*_: bacterial excretion with outflow rate *φ* [19].

Distribution of bacterial strains in pigs was assumed to be homogeneous. Plasma concentration is often used as a surrogate for the concentration at the interaction site, and was therefore used for the bacteria-drug interaction after an IM injection [20]. The value of the carrying capacity *N*_*max*_ was 10^10^. The outflow rate *φ* was taken from published studies with a value of 0.01 [21, 22]. The model was initiated with a composition of ten strains with corresponding growth parameters as described in the previous section. A random selection between 10^6^ and 10^9^ were allocated as initial values of individual strains. The model was first run without drugs to attain the dynamic equilibrium in the absence of selection pressures [18].

Treatment was introduced once equilibrium was attained with the inclusion of ten *E. coli* strains competing in the intestinal flora of a pig, and the first treatment day defined as Day 0. In order to assess the growth dynamics post-treatment, the model was allowed to run for a total of 35 days after the initiation of treatment. This time is referred to as the end of the weaning period, as treatment in Danish pig herds is typically initiated during the first two weeks in the weaning section, where the weaning period lasts for around 7 weeks. Tetracycline dosage was 20 mg/kg and ampicillin was 40 mg/kg. Sequential treatments with 2, 3, and 6 cycling frequency were assessed, where a cycling frequency of 2 refers to the use of each drug for 3 days. To clarify, cycling frequencies could be written in the following manner: 2 as (3 + 3), 3 as (2 + 2 + 2), and 6 as (1 + 1 + 1 + 1 + 1 + 1). These sequential treatments were also assessed in reverse order, i.e. with tetracycline as the first drug, and then with ampicillin as the first drug.

The uncertainties in the estimated PD parameters were assessed by model outputs for 100 repeats, where in each repeat; parameters were randomly selected from a normal distribution of estimated parameters. A simulation envelope from these repetitions with mean value was reported in the graphs for single and combination treatments only. The model was written in R (version 3.0.1 for windows) [14], and all data were also analyzed and plotted using R.

## Results

**Fig. 2:**
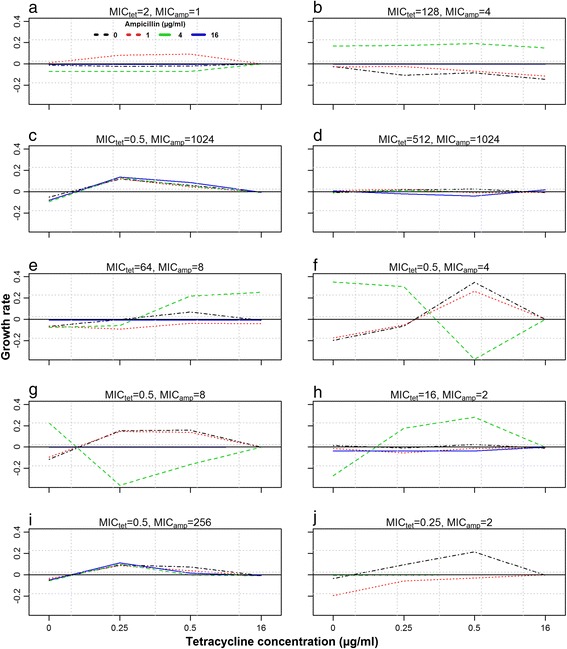
Interaction plots of tetracycline and ampicillin showing the relationship between antimicrobial concentration and the residuals from the fitted combination drug effect model. Each box represents one bacterial strain, with tetracycline concentration on the x-axis and different lines representing ampicillin concentration

Combined effect of tetracycline and ampicillin on net bacterial growth rates of 10 different strains is shown as an interaction plot (Fig. 1). Different lines in each subplot depict the ampicillin concentrations whereas tetracycline concentrations were plotted on x-axis. Complete horizontal lines represent zero tetracycline effect, whereas overlapping lines represent zero ampicillin effect on net bacterial growth rate. This occurred due to high MIC values of strains to tetracycline and ampicillin (512 μg/ml, 1024 μg/ml etc.), as the maximum concentration of both drugs used in growth curves was 16 μg/ml. The estimated model parameters, along with previously estimated parameters (when used as a single antimicrobial) are given in Table 1. The model used existing information only for the strains with MIC values higher than the exposed concentrations (Table 1). There was no consistent residual structure over the interaction between the two drugs when analyzed for the different bacterial strains in the model (Fig. 2), indicating that no interaction occurred.

**Fig. 3:**
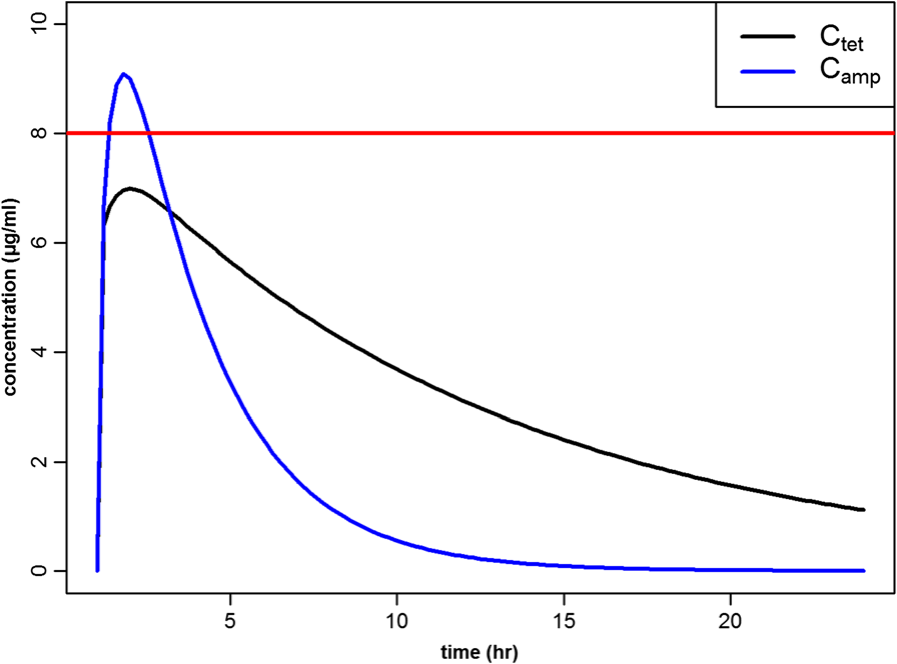
Plasma concentration-time profiles of intramuscular tetracycline (black) and ampicillin (blue) treatments in pigs, based on transfer rates, estimated from the two-compartmental model. The red line represents the cut-off value between susceptible and resistant strains to both tetracycline and ampicillin

Having determined the PD parameters, we proceeded to the pharmacokinetics of tetracycline and ampicillin. As described in the methods section, we simulated plasma concentration-time profiles of the two drugs, based on estimated transfer rates (Fig. 3). The two drugs differed in pharmacokinetic (PK) properties, with ampicillin having a high absorption and elimination rate with large daily peak concentration, and tetracycline having a lower elimination with a smaller peak concentration. The concentration remained mostly below the MIC values of resistant strains (MIC > 16) during the treatment period, giving resistant strains substantial opportunity to outcompete the susceptible ones (Fig. 3).

**Fig. 4:**
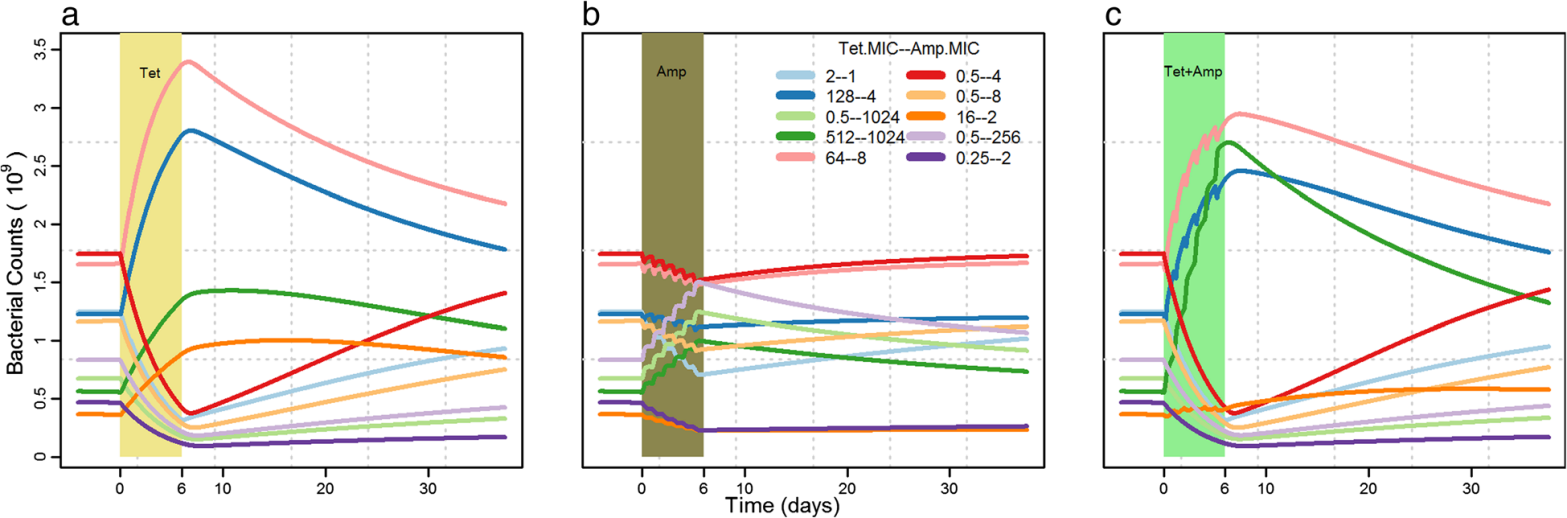
Growth dynamics of ten competing strains before, during and after different antimicrobial treatments. Each line represents a bacterial strain with different combinations of minimum inhibitory concentration (MIC) values to tetracycline and ampicillin. Treatment is represented by the color bar from 0 to 6 days

The estimated growth parameters (Table 1), along with the PK profiles of the two drugs were used in the mathematical model (equation 3) to simulate the growth dynamics of the ten competing strains in a pig under single and dual-antimicrobial treatment of tetracycline and ampicillin (Fig. 4). The treatment period of 6 days is shown by a colored window, with treatment start at Day 0. The time before the treatment window was the period taken by the model to establish dynamic equilibrium between the ten competing strains. Dynamic equilibrium is defined as the growth of multiple strains in a pig, with very small changes over long periods of time. Antimicrobial treatment disturbs the dynamic equilibrium of the system, which takes varying lengths of time to return after the treatment ends. For tetracycline single treatment, three strains resistant to tetracycline (R_T_S_A_) and one strain resistant to both tetracycline and ampicillin (R_T_R_A_) had a clear growth advantage (Fig. 4a).

**Fig. 5:**
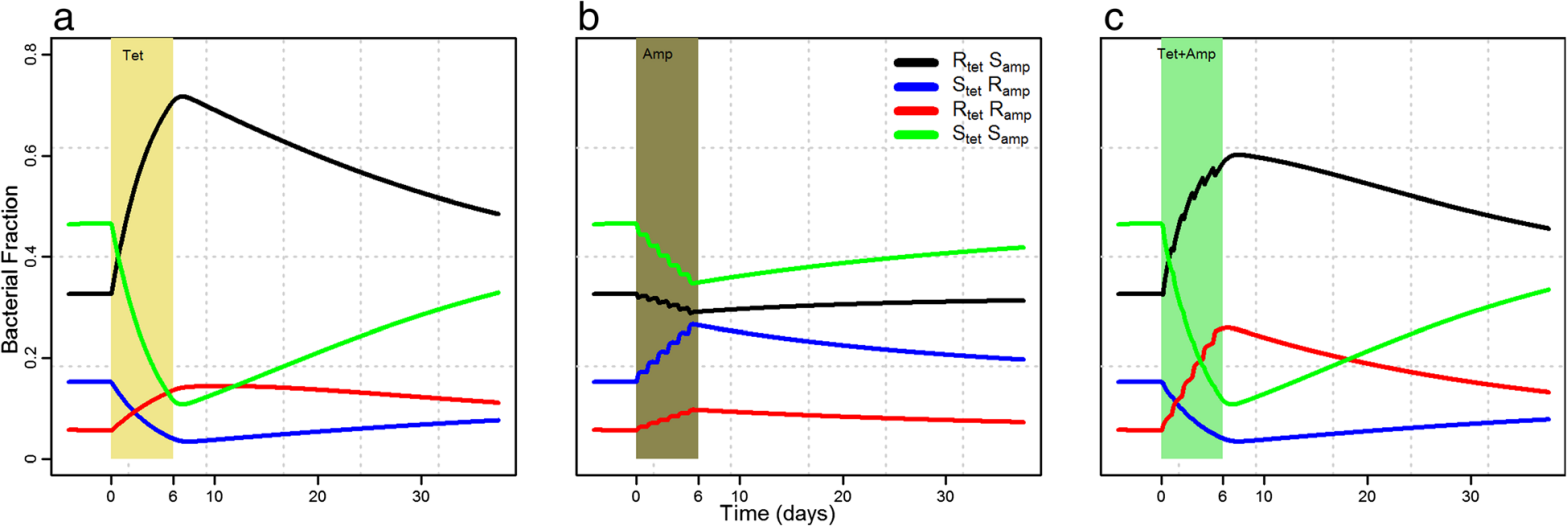
Fraction of total bacterial counts of strains resistant to tetracycline (black), resistant to ampicillin (blue), resistant to both (red), and susceptible to both (green) during and after tetracycline, ampicillin and tetracycline + ampicillin intramuscular treatment. Treatment duration is represented by the color bar

Due to the high level of resistant bacteria at the end of the tetracycline treatment, it took a long time to regain the equilibrium. In the case of ampicillin single treatment, the system was also disturbed but not to a large extent (Fig. 4b), which allowed the system to regain the equilibrium rapidly. The combination treatment looked similar to the tetracycline single treatment except double resistant strains (Fig. 4c, green line) had more growth. When comparing the fraction of bacterial counts in each of the four groups, the double resistant group had more growth advantage than both single treatments (Fig. 5, red lines). For simplification, corresponding resistant fractions were quantified at three time points and are given in Additional file 2: Figure S2.

**Fig. 6:**
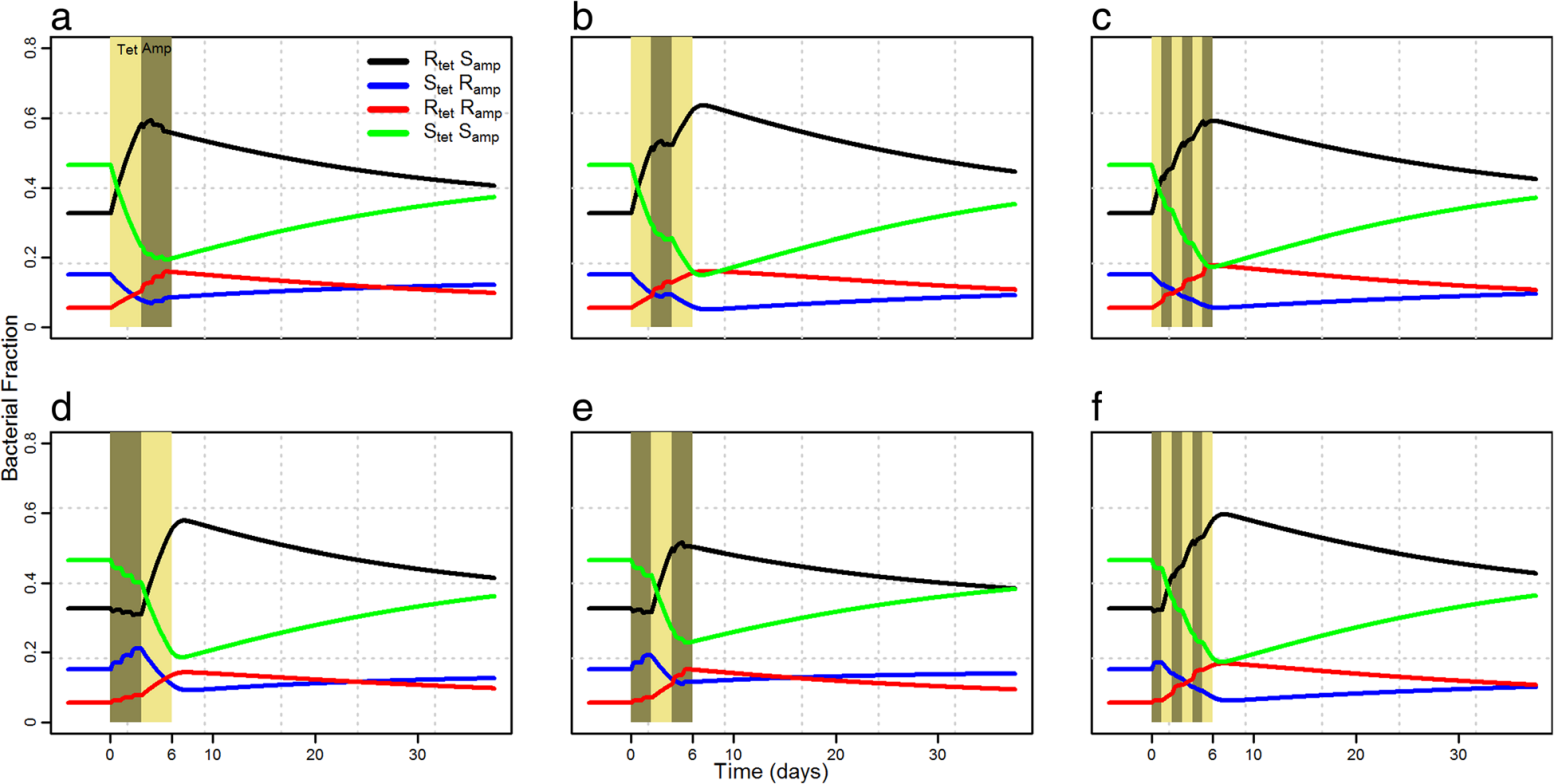
Fraction of total bacterial counts of strains resistant to tetracycline (black), ampicillin (blue) and both (red) during and after sequential treatments of tetracycline and ampicillin. **a**, **b**, **c** Treatment with tetracycline is initiated first. **d**, **e**, **f** Treatment with ampicillin is initiated first

Sequential treatments (Fig. 6) were found to allow double resistant strains (red lines) a quicker return to equilibrium than in combination treatment (Fig. 5c), irrespective of cycling frequency. However, within different sequential treatments, increased cycling frequency did not have a major effect on the growth dynamics of any of the resistant groups (Fig. 6). Moreover, the order of the two drugs in sequential treatments was only of consequence when treating with odd cycling frequency (Fig. 6e) as it resulted in higher tetracycline resistant strains when tetracycline is used first whereas lower when ampicillin were used first. For cycling frequency of 2 (Fig. 6a, d) and 6 (Fig. 6c, f), the growth dynamics of each resistant group were found to be similar in both sequences. Quantifications of resistance at three time points are shown in Additional file 3.

**Fig. 7:**
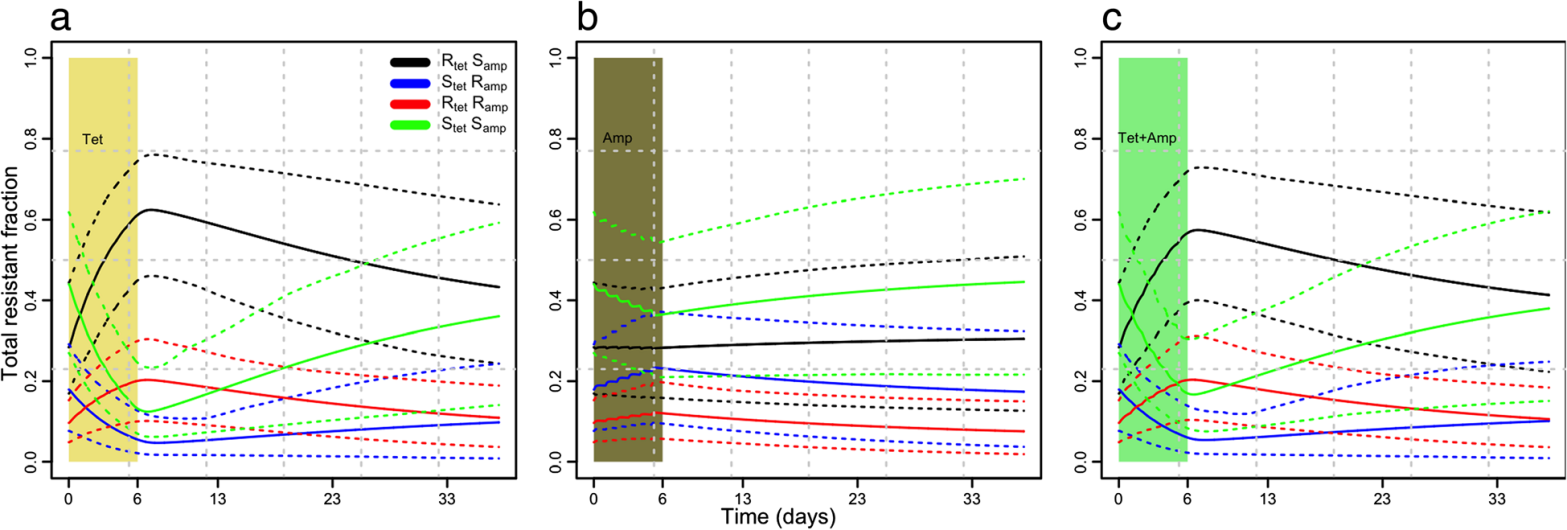
Mean and 95 % simulation envelope from 100 repeats of total resistant fraction in each of four groups. Pharmacodynamic parameters in each repeat were randomly drawn from a normal distribution of estimated parameters

A broad simulation envelope was reported around the mean from 100 repetitions, mainly in the tetracycline-resistant group, which showed large uncertainty in growth parameters of the tetracycline concentration effect (Fig. 7).

## Discussion

Antimicrobial pressure plays a central role in the emergence of antimicrobial-resistant bacteria. In this paper, we have presented a model evaluating the important factors in multidrug treatment which are responsible for the emergence of antimicrobial resistance. The model evaluated the role of combination treatment and sequential treatment in preventing the growth of resistant strains. Firstly, the model showed that combination treatment provides an extra advantage for the double resistant strains to grow and outcompete the susceptible strains when compared to sequential treatments. This is in accordance with the guidelines for improving antibiotic use from the Infectious Disease Society of America and the Society for Healthcare Epidemiology [23]. Secondly, the model showed that the cycling frequency in sequential treatments is not very important in suppressing the growth of resistant strains, whereas the order of two drugs is influential when using an odd value of cycling frequency.

Multidrug treatments have been used to treat infections caused by many important bacterial pathogens [4, 6]. At the same time, the emergence and growth of antimicrobial resistance in commensal bacteria is an important concern [6, 24]. High levels of resistance are generally considered to be strongly correlated with selection pressures from antimicrobial use. The use of multiple drug treatments could boost the growth of resistant bacteria due to large antimicrobial pressure [6]. Careful investigation of multidrug treatments could provide better treatment strategies that would help to reduce the selection and growth of resistant strains. Many models have been proposed to evaluate the treatment factors that affect the growth of resistant strains [20, 21, 25–29]. Many of these studies used single drug exposure, and some used multidrug exposure to assess the effect on the emergence of resistance. Here, we have used a mathematical model to investigate the effect of both combination and sequential treatments on the level of resistance in a scenario of multiple strains, irrespective of the infection status.

The model produced realistic changes in antimicrobial pressure of both tetracycline and ampicillin based on the transfer rates of the two-compartmental model fitted to the plasma concentration-time profiles. In contrast to previous studies, realistic plasma concentration-time profiles were used in the model (equation 2) to simulate a multidrug effect on growth [21, 28]. Moreover, we have included multiple strains with different antimicrobial susceptibility profiles in the model, whereas previous studies evaluated multidrug treatments for a single or small number of clinical bacterial strains only [5, 28].

When considering the bacterial growth in a pig, one could argue that high resistance is already established prior to treatment. Therefore, we have not considered the role of mutation and conjugation mechanisms in the growth of resistant strains. As plasma concentrations are considered an appropriate surrogate marker in most PK studies and it is difficult to measure drug concentration in the intestine, we have used plasma concentration as an alternative to drug concentration at the interaction site [30]. Both ampicillin and tetracycline are mainly eliminated by the kidneys, meaning that a much less amount than the dose of both drugs reach the intestines from the blood and could produce different results and must be accounted for further studies. A single isolated pig was considered in the model, with excretion of bacteria from the pig represented by outflow rate (equation 3). The outflow rate was kept constant, but it could be varied dependent on the disease status. The bacterial growth rate in the absence of a drug (*α*_max_) varied for different strains and was previously considered to play a role in growth dynamics when comparing multidrug treatments. However, it was assessed by resetting the *α*_max_to the same value for all strains in the model and was found to have no apparent difference (data not shown).

Large uncertainties in PD parameters (reflected in Fig. 7) were due to a small number of data points between maximum and zero growth. These uncertainties could be reduced by performing in vitro growth curves under narrow ranges of antimicrobial concentrations. Limiting growth factors in the model (equation 2) reflects the in vivo situation where multiple strains coexist in a pig under a state of dynamic equilibrium. Our conclusions were mainly based on simulations of the model (equation 3), but in future, experimental studies could be performed to support our model simulations and conclusion.

We examined the multidrug treatments by assessing the effect on the growth of multiple strains in a pig. The goal was to compare the efficacy of combination treatment with both single and sequential treatments in suppressing the growth of resistant strains. The effect of combination treatment was similar to the effect of single tetracycline treatment except there was more growth observed in the double resistant group (Fig. 5).

Combination treatment with tetracycline and the antagonistic drug ciprofloxacin has previously been shown to give an advantage of sensitive strains over tetracycline resistant *E. coli* [8]. β-lactam drugs and tetracycline are normally considered antagonistic because tetracycline works by inhibiting growth, while β-lactam only works on growing cells. In the current study, we could not find indications that such an interaction was taking place. Possible interpretations could be that there was no interaction between tetracycline and ampicillin or that the interaction was dependent upon a complex combination of MIC and drug concentrations. In other words, the interaction plot from the current study suggested that the two drugs with the MIC and concentration distributions tested had a pharmacodynamic effect independent of the other drug, when used in combination.

## Conclusions

In summary, sequential treatment was more effective in preventing the growth of resistant strains when compared to the combination treatment. The cycling frequency did not play a role in suppressing the growth of resistant strains, but the specific order of the two antimicrobials did. Uneven numbers of two drugs (such as ampicillin + tetracycline + ampicillin for 2 days each) in sequential treatment could be an important consideration when designing multidrug treatment strategies. These predictions could be used to redesign multidrug treatment strategies not only for IM treatment in pigs, but also for other dosing routes.

## Abbreviations

CFU, Colony forming units; *E. coli*, *Escherichia coli;* IM, Intramuscular; MIC, Minimum inhibitory concentration; OD, Optical density; PD, Pharmacodynamic; PK, Pharmacokinetic.

## Additional files

Additional file 1: Supplementary Figure S1. (TIFF 88 kb) Additional file 2: Supplementary Figure S2. (TIFF 131 kb) Additional file 3: Data used to estimate growth rates. (CSV 1594 kb)
