# Supplementary figures and images for: Multistrain models predict sequential multidrug treatment strategies to result in less antimicrobial resistance than combination treatment
Source: BMC Microbiol. 2016 Jun 23;16:118. doi: 10.1186/s12866-016-0724-5 (PMC4917987; doi:10.1186/s12866-016-0724-5)

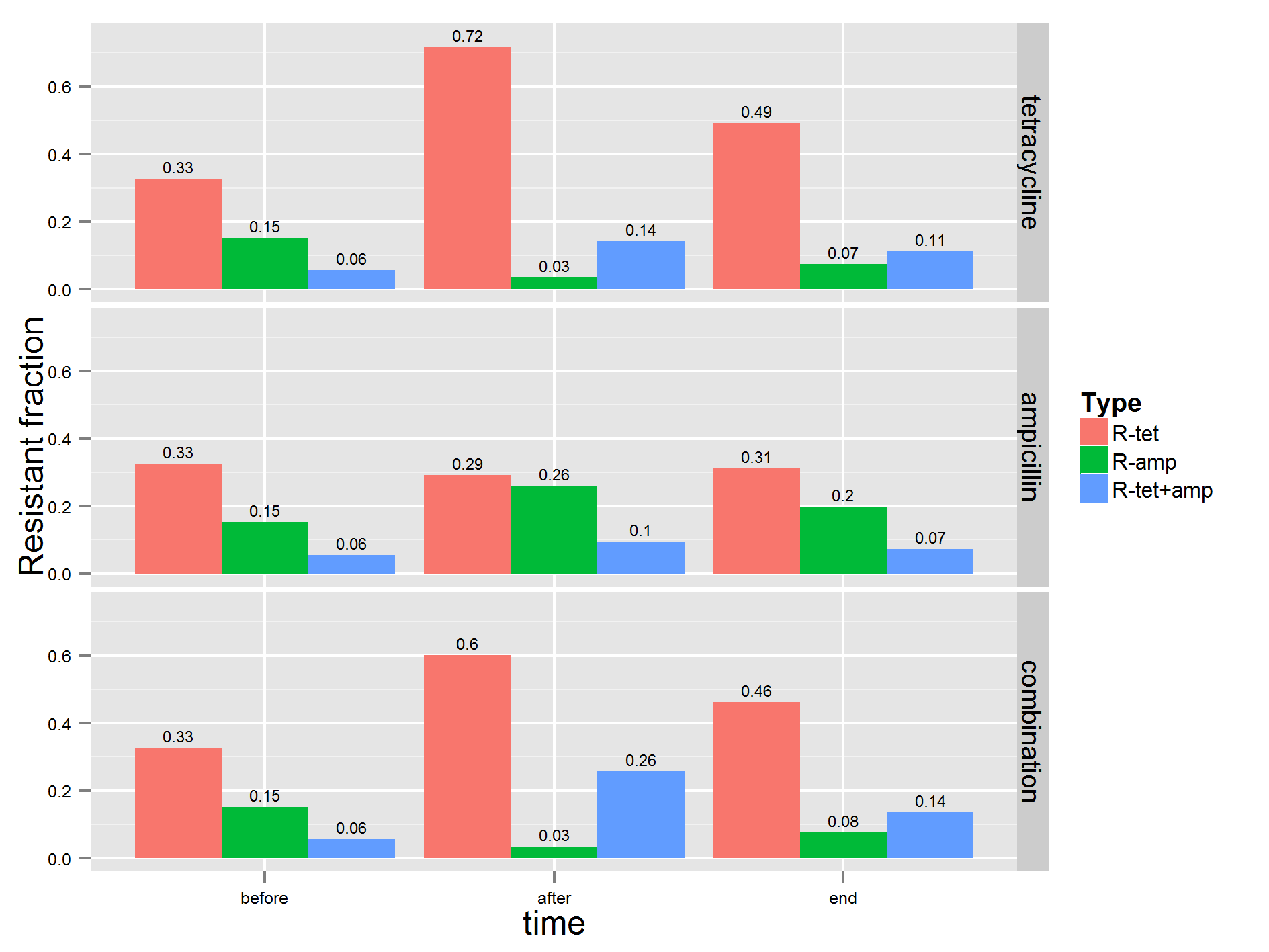

Supplement: Additional file 1: — Supplementary Figure S1. (TIFF 88 kb) [file 12866_2016_724_MOESM1_ESM.tiff]

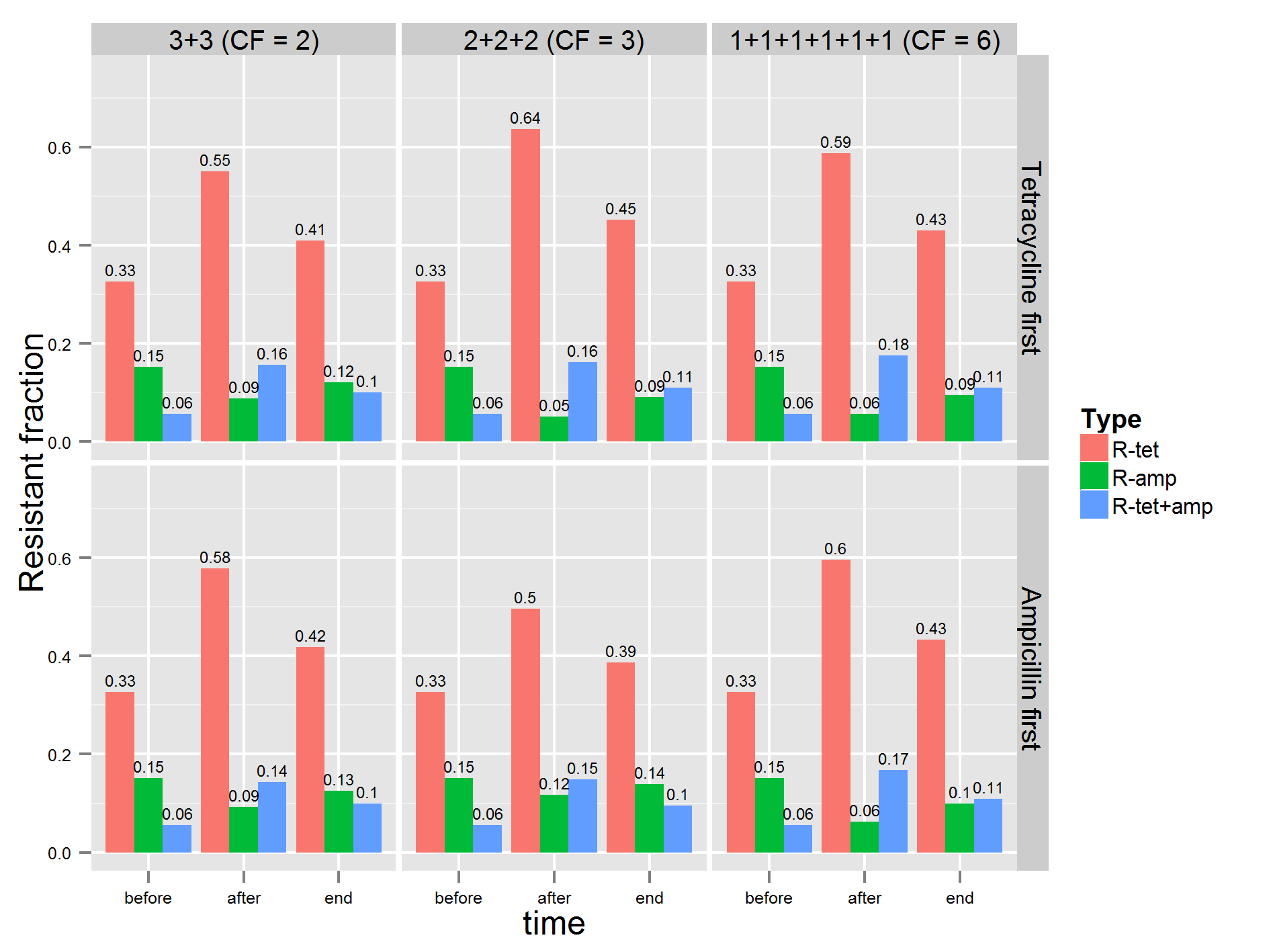

Supplement: Additional file 2: — Supplementary Figure S2. (TIFF 131 kb) [file 12866_2016_724_MOESM2_ESM.tiff]
